# Differential expression of microRNAs among cell populations in the regenerating adult mouse olfactory epithelium

**DOI:** 10.1371/journal.pone.0187576

**Published:** 2017-11-06

**Authors:** Sarah Kurtenbach, Wen Ding, Garrett M. Goss, Joshua M. Hare, Bradley J. Goldstein, Lina A. Shehadeh

**Affiliations:** 1 Interdisciplinary Stem Cell Institute, University of Miami Leonard M. Miller School of Medicine, Miami, Florida, United States of America; 2 Department of Otolaryngology, University of Miami Leonard M. Miller School of Medicine, Miami, Florida, United States of America; 3 Department of Molecular and Cellular Pharmacology, University of Miami Leonard M. Miller School of Medicine, Miami, Florida, United States of America; 4 Department of Medicine, Division of Cardiology, University of Miami Leonard M. Miller School of Medicine, Miami, Florida, United States of America; 5 Vascular Biology Institute, University of Miami Leonard M. Miller School of Medicine, Miami, Florida, United States of America; University of Massachusetts Medical School, UNITED STATES

## Abstract

Despite a robust capacity for adult neurogenesis in the olfactory epithelium (OE), olfactory sensory losses are common. Identification of mechanisms regulating adult OE neurogenesis is, therefore, of interest. MicroRNAs (miRNAs) are broadly important in regulating vertebrate neurodevelopment, and are required in embryonic olfactory differentiation. We report here that a panel of miRNAs is differentially expressed by either progenitor or progeny cells in the regenerating mouse OE. Progenitor cells were purified from lesioned OE based on c-Kit expression, and miRNA expression was assayed in c-Kit (+) and c-Kit (-) cell populations. 28 miRNAs were significantly downregulated by at least 4 fold in the c-Kit (+) fraction, which marks the globose basal progenitor cell population. In addition, 10 miRNAs were upregulated in these basal cells. MiR-486, the most strongly downregulated miRNA identified, was further characterized to verify results. MiR-486 expression was confirmed in the c-Kit (-) OE layers using *in situ* hybridization. As a functional assay, over-expression of miR-486 in purified c-Kit (+) basal cell cultures resulted in a reduction in neurogenesis, consistent with a possible negative feedback regulatory model. Our data provide new insights regarding miRNA expression and function during adult OE neurogenesis, and identify candidate miRNAs warranting further study.

## Introduction

MicroRNAs (miRNAs) are major posttranscriptional regulators of gene expression [1]. The first miRNA was identified in *C*. *elegans* in 1993 [2], and the importance of mammalian miRNAs is now widely recognized in development and disease. By base-pairing with complementary sites in their target messenger RNAs (mRNAs), miRNAs control the repression of mRNAs, primarily through mRNA destabilization [3–5]. With each miRNA capable of targeting mRNAs of hundreds of genes, and well over half of the human transcriptome harboring conserved miRNA binding sites [6], miRNAs are predicted to impact many key mammalian processes, including neuronal differentiation. Studies of invertebrate neurogenesis have revealed roles for specific miRNAs in neurodevelopment. For instance, miR-273 and lsy-6 regulate the expression of taste receptors in *C*. *elegans* chemosensory neurons [7, 8], while *Drosophila* miR-7 regulates photoreceptor cell differentiation [9].

In accordance with these findings in invertebrates, studies of murine olfactory neurogenesis during embryonic development have also identified a requirement for functional miRNAs [10]. Specifically, conditional disruption of Dicer function in embryonic olfactory progenitors, which prevents miRNA production, resulted in severe defects in neurogenesis. Because these manipulations led to prenatal lethality, further insights regarding miRNAs in the adult mammalian olfactory epithelium (OE) with this approach were limited. However, the olfactory system provides a unique model for examining mechanisms involved in adult neurogenesis [11, 12]. Olfactory sensory neurons tend to have a lifespan in the order of months [13], although there is considerable variation. The neurons reside in an epithelium in contact with the nasal airspace and, under normal homeostatic conditions, are replaced continually from stem and progenitor cells in the basal layers [12, 14–18]. Basal cells can also produce non-neuronal cell populations, including apical sustentacular and microvillar cells, as well as Bowman’s glands [12, 17, 19], especially after severe OE damage. By manipulating the status of the OE in mice using experimental injury models, adult neurogenesis and its regulatory mechanisms are amenable to *in vivo* studies. Injury models include olfactory bulbectomy or nerve section [20, 21], which damage only neurons and induce neurogenesis, or direct/chemical lesion models [15, 22–24], which cause loss of sustentacular cells, neurons and some basal cells. Of these, the methimazole lesion model is simple, reliable, well characterized, and has been useful for several recent studies of adult OE reconstitution [12, 18, 23]. Adult OE neurogenesis is also of clinical importance, since common acquired sensory losses (anosmias) appear to be associated with a histologic picture of neurogenic exhaustion [25].

Given the importance of miRNAs during embryonic development of the OE, it is logical to expect ongoing roles for miRNAs in adult olfactory neurogenesis and tissue homeostasis. While mRNA profiling of adult OE populations has been reported [26–30], the differential expression of miRNAs between the basal stem and progenitor cells and their differentiating progeny fractions in the regenerating OE has not been specifically investigated. Accordingly, we sought to purify OE cells for miRNA profiling. Here, we have isolated progenitor populations from regenerating mouse OE based on c-Kit expression [18, 30, 31]. We present global miRNA profiling in progenitor c-Kit (+) versus non-progenitor c-Kit (-) cell fractions in the regenerating adult mouse OE. Here, we show that several miRNAs are selectively enriched in progenitor or non-progenitor cell fractions in the regenerating adult mouse OE. We found that miR-486 was enriched in the non-progenitor fraction and its forced over-expression *in vitro* in c-Kit (+) progenitor globose basal cells (GBCs) has an inhibitory effect on mature neuron production. To our knowledge, this is the first report to specifically address miRNAs in mature mammalian OE maintenance.

## Materials and methods

### Animals

All experimental procedures were approved by the University of Miami Institutional Animal Care and Use Committee, and were performed in full compliance with the NIH Guidelines for the Care and Use of Laboratory Animals. Mice were housed in a barrier facility, with ad libitum food and water and a 12-hour light-dark cycle. 6–12 week old C57BL6 mice (Jackson Lab, Bar Harbor, ME) were used for tissue harvest for histology, cell purification for RNA preparation, or cell culture. Methimazole (Sigma, St. Louis) was dissolved in phosphate buffered saline (PBS) and injected intraperitoneally at 50 μg/g body weight 10 days prior to sacrifice, to induce olfactory lesion and regeneration [23]. For euthanasia, mice were anesthetized with an over dose of ketamine-xylazine intraperitoneally, prior to decapitation and tissue harvest.

### Cell isolation

10 days following methimazole lesion, adult mice were deeply anesthetized, decapitated, and nasal tissue was harvested, enzymatically dissociated into a single cell suspension, and washed. Immunomagnetic selection was performed using the EasySep Mouse APC Positive Selection kit (Stemcell Technologies, Vancouver, BC) to label cells with APC-conjugated antibody against c-Kit (eBiosciences 17–1171; RRID:AB_469430), as previously described [31].

### Microarrays and bioinformatics

Total RNA from sorted c-Kit (+) and (-) fractions was extracted using Quick-RNA MicroPrep kit (Zymo Research, Irvine, CA) to yield a 260–280 nm absorbance ratio of 2.0. RNA concentration and integrity was confirmed using an Agilent Bioanalyzer. Briefly, 500ng total RNA was prepared using the Affymetrix FlashTag Biotin HSR RNA Labeling Kit (Part# 901910) according to manufacturer’s protocol (3 samples per group; 2 groups per experiment). Fragmented and labeled products were hybridized to the Affymetrix GeneChip miRNA 4.0 Array. Washing and staining of arrays took place on the GeneChip Fluidics Station 450 and scanning was done on the GeneChip Scanner 3000 7G system. Expression ratios were calculated as the power-2 exponential of the log2 differences. The acceptance criteria for gene array expression changes was a minimum 2 fold change in log2 (equivalent to 4 fold) and a one-way Analysis of Variance (ANOVA) t-test with Benjamini-Hochberg multiple testing correction p-value of <0.05. All microarray raw files were submitted to the NCBI Gene Expression Omnibus (GEO) database (GSE104185).

### Quantitative RT-PCR

For miRNA quantification, reverse transcription was performed using miRNA-specific Taqman primers, and cDNA was amplified using Taqman primers against the mature miRNA strands. Data was analyzed using the RQ Manager 1.2 from Applied Biosystems, CA. TaqMan assays were run in duplicate for each miRNA in each sample (n = at least 3 biological replicates/condition) and all miRNA levels were normalized to endogenous Sno RNA.

### *In situ* hybridization

Adult mice were deeply anesthetized and perfused with cold PBS followed by 4% paraformaldehyde in PBS. Nasal tissue was isolated, post-fixed, processed in 30% sucrose with 250 mM EDTA in DEPC-PBS, cryosectioned at 10 μm, collected on Superfrost Plus slides (VWR, Radnor, PA), and stored at -80°C. *In situ* hybridization was performed as previously described [32]. Briefly, slides were pretreated with post-fix followed by Proteinase K (20 μg/ml) digestion for 10 min, acetylation for 10 min, and PBS rinses. Pre-hyridization was performed for 1 hour at room temperature with hybridization buffer. 3’ and 5’-double-digoxigenin labeled LNA probes for miR-486-5p (CTCGGGGCAGCTCAGTACAGGA), or a scrambled negative control (GTGTAACACGTCTATACGCCCA), were obtained from Exiqon (Woburn, MA). Probes were diluted in hybridization buffer to 0.1 μM, denatured, then hybridized overnight at 60°C. Slides were then washed in 0.2x SSC twice for 30 min at 65°C, then PBS for 5 min, and then processed for anti-DIG-AP. Slides were blocked in PBS with 5% nonfat milk, 4% bovine serum albumin, 5% normal goat serum, and 0.1% Tween 20 for 30 min, incubated with anti-DIG-AP (#11093274910 Roche, Sigma, St. Louis) at 1:250 for 1 hour at room temperature, washed and visualized with NBT/BCIP reaction. Slides were rinsed in PBS with 1 mM EDTA, pH 5.5, then cover slipped with Aquamount (VWR). Images were digitally acquired on a Zeiss Axiophot microscope with phase contrast optics and assembled using ImageJ software.

### Cell culture assays

Globose basal cells were prepared for culture as described previously [31]. The c-Kit (+) cell fraction was isolated by immunomagnetic selection as described above. Cells were cultured on Vitronectin-coated coverslips in complete NeuroCult medium (Stemcell Technologies) overnight. Cells were transfected 24 hours later with either 10 nM miR-486 or scrambled control miRNA from Exiqon (Woburn, MA) using Lipofectamine 2000 transfection reagent (Thermo Fisher Scientific) according to manufacturer’s instructions. Cells were then processed for further analyses 48 hours post transfection. EdU was added at 3μM 1 hour prior to fixation and visualized by Click chemistry reaction, per manufacturer’s instructions (Invitrogen).

### Immunochemistry

For immunocytochemistry or immunohistochemistry, cultures were fixed with 4% paraformaldehyde in PBS for 15 min RT and washed in PBS for 5 minutes, or cryosections were prepared from paraformaldehyde perfused mice [18]. Blocking was performed using PBS with 10% normal goat serum (Vector Labs), 4% bovine serum albumin (BSA; Sigma) and 0.1% Triton X-100 (Sigma) for 30–60 minutes at room temperature. Primary antibodies (listed in Table 1) were diluted in the same blocking buffer and applied for 2 hours at room temperature, followed by three 5 min PBS washes. Appropriate Alex488 or Alexa 594 conjugated secondary antibodies (Jackson ImmunoResearch) were applied for 60 minutes at room temperature, followed by three 5 minute PBS washes. Cells were mounted with Vectashield containing 4,6-diamidino-2-phenylindole (DAPI; Vector). For quantification of stained cultures, cells were analyzed for immunofluorescent label and DAPI nuclear staining using the EVOS FL Auto system (Thermo Fisher Scientific). Cells were further analyzed using an Olympus IX81 epifluorescent microscope. A total of n = 8 coverslips from 3 independent culture experiments were counted. For each experiment, the percentage of TUJ1-positive neurons (cells with round soma and neurite-like processes) from miR-486 or scrambled- transfected cells from each coverslip was normalized to the averaged values for the scramble control. Statistical analyses were performed using a Student’s t test with Prism 7 (Graphpad).

**Table 1:** Antibody reagents.

Antibody	Supplier	RRID	Concentration
mouse Tuj1; anti-neuron-specific β tubulin	Covance, #MMS-435P	AB_10063408	1:500
rabbit anti-c-KIT	Cell Signaling Technology #3074	AB_10829442	1:30
rat anti-Sox2	eBioscience #14–9811	AB_11219471	1:100
rabbit anti-OAZ	Bethyl Labs #A304-018A	AB_2620366	1:1000
rabbit anti-Cytokeratin 5	Abcam #ab24647,	AB_448212	1:1000
goat anti-olfactory marker protein	WAKO #019–22291	AB_664696	1:1000

Antibody reagents used in the present experiments are listed. Research Resource Identifiers (RRIDs) are provided; all antibodies have been previously validated.

## Results

### Identification of differential miRNA expression in OE cell populations

**Fig 1:**
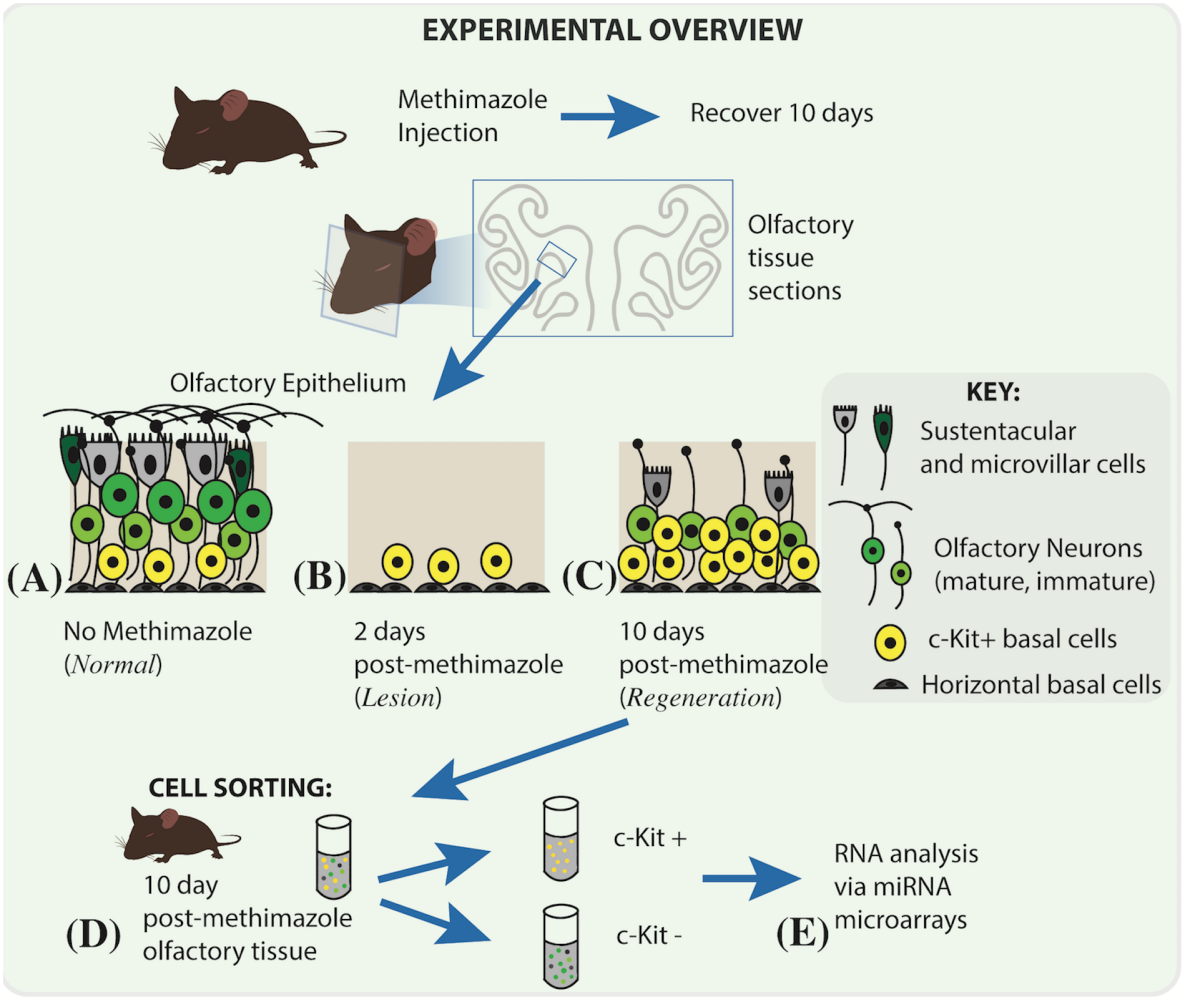
Schematic of experimental strategy to identify differential miRNA expression in olfactory epithelial cells. (A) The olfactory epithelium, lining portions of the mouse nasal cavity, contains basal stem and progenitor cells, immature differentiating olfactory receptor neurons, mature neurons, sustentacular cells, and microvillar cells. (B) Following experimental lesion with methimazole, spared basal globose cells remain, and their expansion and differentiation (C) lead to rapid epithelial reconstitution. (D) Olfactory epithelial cells harvested 10 days following lesion are enriched with basal progenitors marked by surface expression of c-Kit. The c-Kit (+) basal cells were isolated by immunoselection, and total RNA was purified from the c-Kit (+) fraction or the c-Kit (-) fraction was analyzed by miRNA microarrays.

To identify miRNAs selectively enriched in different OE cell populations, we utilized an unbiased global microarray approach (Fig 1). In previous studies Dicer, an enzyme required for mature miRNA production, was conditionally disrupted in OE progenitors using Foxg1-Cre/ Dicer^fl/fl^ mice and resulted in a failure of olfactory neurogenesis, along with widespread defects and prenatal death [10]. We reasoned that during adult olfactory tissue regeneration, the progenitor cells active in producing new olfactory neurons are similarly subject to regulation by specific miRNAs. Using the mouse methimazole lesion-regeneration model [23], we obtained OE tissue actively regenerating after experimental injury, enriched for neurogenic progenitor cells (Fig 1). We purified OE progenitor cells based on their expression of the receptor tyrosine kinase c-Kit [18, 30, 33], as previously described [31]. MiRNAs expressed in the purified c-Kit (+) progenitor cell fraction (n = 3 biologic replicates, 3 mice per preparation) and in the c-Kit (-) fraction were then assessed using miRNA microarrays. Importantly, RNA was purified acutely upon isolating the cells from tissue, without an intervening culture step, in an effort to identify miRNAs expressed during OE regeneration in vivo.

**Fig 2:**
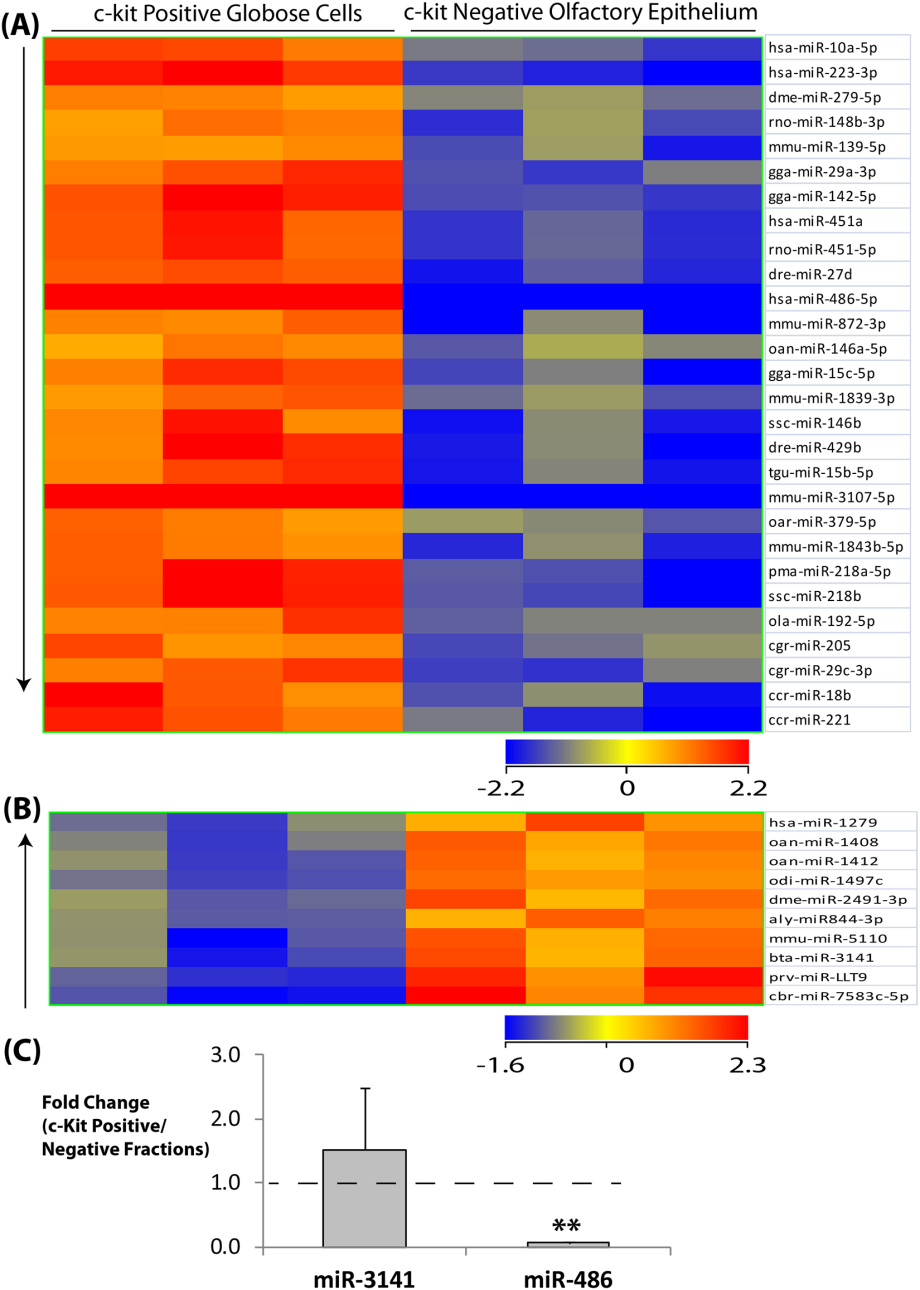
Differential miRNA expession in cell populations of the olfactory epithelium. Heatmap shows microarray results of top 28 miRNAs most downregulated (A) and top 10 miRNAs most upregulated (B) in c-Kit (+) versus c-Kit (-) fractions of mouse olfactory epithelium 10 days post olfactory lesion. N = 3 per group. Criteria used for selection is at least 4 fold change in transcript expression with p<0.05 based on t-test with Benjamini-Hochberg multiple testing correction. (C) QPCR validation shows miR-486 is 17 fold downregulated in c-Kit (+) versus (-) fractions of mouse olfactory epithelium 10 days post nasal injury. N = 3 per group. Data are Mean ± S.E.M. p = 0.001 based on t-test using delta Cts.

Using this approach, we hypothesized that miRNAs active in adult OE neurogenesis would be detectable, and that miRNAs selectively enriched in the c-Kit (+) or the c-Kit (-) populations should represent candidates of particular interest. Although the c-Kit (-) fraction is rich in neurons, it also contains sustentacular cells and a small number of horizontal basal cells, Bowman’s gland/duct cells, and possibly some underlying stromal cells (Fig 1). Our results indicated that 28 miRNAs are significantly downregulated by at least 4 fold in c-Kit (+) versus c-Kit (-) mouse OE cells (Fig 2A, Table 2). Additionally, we identified 10 miRNAs upregulated at least 4 fold in the c-Kit (+) basal cell fraction (Fig 2B, Table 3). Two miRNAs were selected for validation by qPCR (Fig 2C): miR-3141, enriched in basal cells, and miR-486, enriched in the c-Kit (-) fraction. While miR-3141 expression trended towards enrichment in c-Kit (+) cells but was variable among qPCR samples, miR-486 was significantly downregulated in the basal cells, and was therefore selected for further study.

**Table 2:** MiRNAs differentially downregulated in c-Kit (+ve) mouse globose cells post injury.

Transcript ID	Fold Change	Corrected p-value
hsa-miR-486-5p	-46.19	0.0033
mmu-miR-3107-5p	-45.10	0.0033
hsa-miR-223-3p	-17.83	0.0127
ssc-miR-218b	-14.24	0.0299
pma-miR-218a-5p	-13.63	0.0338
dre-miR-429b	-10.93	0.0464
gga-miR-142-5p	-10.71	0.0034
ccr-miR-221	-9.47	0.0262
gga-miR-15c-5p	-9.34	0.0370
hsa-miR-451a	-9.10	0.0089
rno-miR-451-5p	-9.04	0.0087
dre-miR-27d	-8.92	0.0037
tgu-miR-15b-5p	-8.90	0.0262
mmu-miR-872-3p	-8.64	0.0467
ccr-miR-18b	-8.11	0.0448
ssc-miR-146b	-8.04	0.0464
gga-miR-29a-3p	-7.45	0.0122
cgr-miR-29c-3p	-7.40	0.0122
hsa-miR-10a-5p	-6.97	0.0100
mmu-miR-1843b-5p	-6.50	0.0262
ola-miR-192-5p	-5.46	0.0153
rno-miR-148b-3p	-5.37	0.0306
mmu-miR-1839-3p	-5.27	0.0191
cgr-miR-205	-5.27	0.0250
mmu-miR-139-5p	-5.09	0.0362
oar-miR-379-5p	-4.61	0.0172
dme-miR-279-5p	-4.08	0.0087
oan-miR-146a-5p	-4.02	0.0263

Microarray analysis shows that 28 miRNAs are significantly downregulated by at least 4 fold in c-Kit (+) versus c-Kit (-) mouse OE cells 10 days post nasal injury. N = 3 mice/arrays per group. P<0.05 based on t-test with Benjamini-Hochberg multiple testing correction.

**Table 3:** MiRNAs differentially upregulated in c-Kit (+ve) mouse globose cells post injury.

Transcript ID	Fold Change	Corrected p-value
aly-miR844-3p	4.08	0.0269
oan-miR-1412	4.25	0.0292
dme-miR-2491-3p	4.30	0.0467
hsa-miR-1279	4.32	0.0467
oan-miR-1408	4.40	0.0262
odi-miR-1497c	4.49	0.0087
mmu-miR-5110	5.05	0.0469
bta-miR-3141	5.10	0.0482
prv-miR-LLT9	7.78	0.0293
cbr-miR-7583c-5p	8.84	0.0257

Microarray analysis shows that 10 miRNAs are significantly upregulated by at least 4 fold in c-Kit (+) versus c-Kit (-) mouse globose cells 10 days post nasal injury. N = 3 mice/arrays per group. P<0.05 based on t-test with Benjamini-Hochberg multiple testing correction.

### MiR-486 is selectively expressed in olfactory neurons

**Fig 3:**
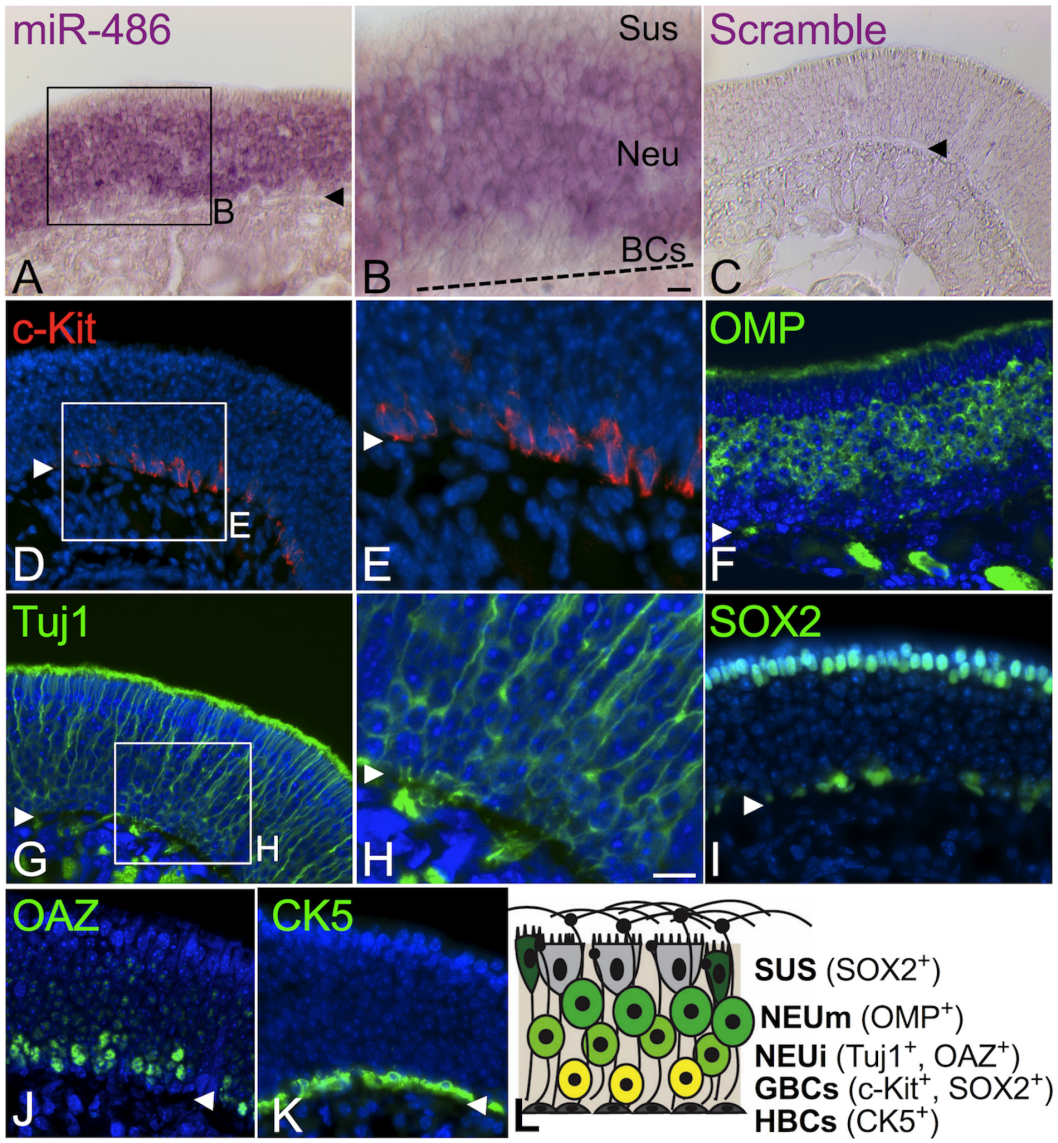
Confirmation by *in situ* hybridization for miR-486 enrichment in the c-Kit (-) population. MiR-486, identified as highly enriched in the c-Kit (-) cell fraction from regenerating olfactory epithelium, was chosen for further study to confirm the microarray findings. *In situ* hybridization was performed on normal adult mouse olfactory epithelium tissue sections; a miR-486 probe (A, B) reveals expression by cells throughout the neuronal layers (Neu), with little signal in the underlying lamina propria. At high magnification (B) an absence of signal in the c-Kit (+) basal cell layers (BCs) or in the apical sustentacular cell layers (Sus) is evident. (C) Hybridization using a scrambled control probe shows no signal. (D-K) Staining with cell type-specific markers was performed to define the OE cell populations. Antibody against c-Kit (D, E) labels only basal cells slightly above the basal lamina; antibody against OMP (F) labels mature neurons in the mid to upper portions of the OE; antibody against Tuj1 (G, H) labels the somata of immature neurons just above the basal cell layers, as well as dendrites extending apically; antibody against SOX2 (I) labels nuclei of a subset of basal cells near the basal lamina and also the sustentacular cell nuclei at the top of the epithelium; antibody against OAZ (J) localizes to nuclei of late GBCs and nascent neurons in deep layers of the OE; antibody against CK5 (K) labels HBCs along the basal lamina. (L) Cell layers and the markers used here are summarized schematically. Arrowheads or dashed line (in B) mark basal lamina; NEUm = mature neurons; NEUi = immature neurons; scale bar in B = 10 μm, in H = 20 μm.

Our cell preparation and sorting protocol was designed to collect OE cells, rather than stromal or non-epithelial components, by relying on dispase to release the OE from the basal lamina. However, to verify that miRNAs found to be enriched in the c-Kit (-) fraction are indeed expressed in OE populations, we performed RNA in situ hybridization. Using a probe for miR-486, the most strongly downregulated miRNA identified in our arrays, we found that miR-486 expression localized selectively to the olfactory neuron layers of the OE in normal unlesioned adult mouse nasal tissue sections (Fig 3A). As expected, at high magnification miR-486 expression was noted to be absent from the basal cell layers, where c-Kit (+) cells are localized, and was also found to be absent from the apical sustentacular cells layer (Fig 3B). No signal was detected in the lamina propria. Hybridization using a scrambled negative control probe produced an appropriate absence of signal (Fig 3C). The position of the c-Kit (+) basal cells was verified immunohistochemically using a previously validated anti-c-Kit antibody [18] (Fig 3D and 3E). Comparison of the staining patterns from Fig 3A and 3B versus Fig 3D and 3E indicates that in the OE miR-486 is expressed in the neuronal layers apical to the c-Kit (+) basal cells.

The phenotypes of the cell layers comprising the unlesioned adult mouse OE are identifiable using a panel of cell type-specific reagents (Fig 3F–3I). Labeling tissue sections using these markers provides further context for the assessment of the miR-486 labeling pattern. For instance, the fully mature olfactory neurons reside in multiple layers in the middle and upper regions of the OE and are labeled by antibody against Olfactory Marker Protein (OMP) (Fig 3F) [15, 34]. Immature or nascent neurons are labeled by antibody Tuj1 [22] or by expression of the transcription factor OAZ [35] (Fig 3G and 3J). OAZ is a highly specific neuron lineage marker, as it functions to regulate olfactory neuron differentiation via interactions with OE transcription factors and SMADs. The transcription factor SOX2 marks a subset of multipotent GBCs and also localizes strongly to the nuclei of sustentacular cells, situated apically in the OE (Fig 3I) [36, 37]. Finally, the reserve horizontal basal cells (HBCs) are identifiable by expression of cytokeratin 5, immediately above the basal lamina (Fig 3K) [38]. OE histology and cell type-specific markers used here are summarized schematically (Fig 3L). The observed expression of miR-486 in the immature and mature olfactory neuron layers is consistent with our microarray and qPCR results, providing validation of these data sets.

### Over-expression of miR-486 in olfactory basal cell-derived cultures reduces neurogenesis

**Fig 4:**
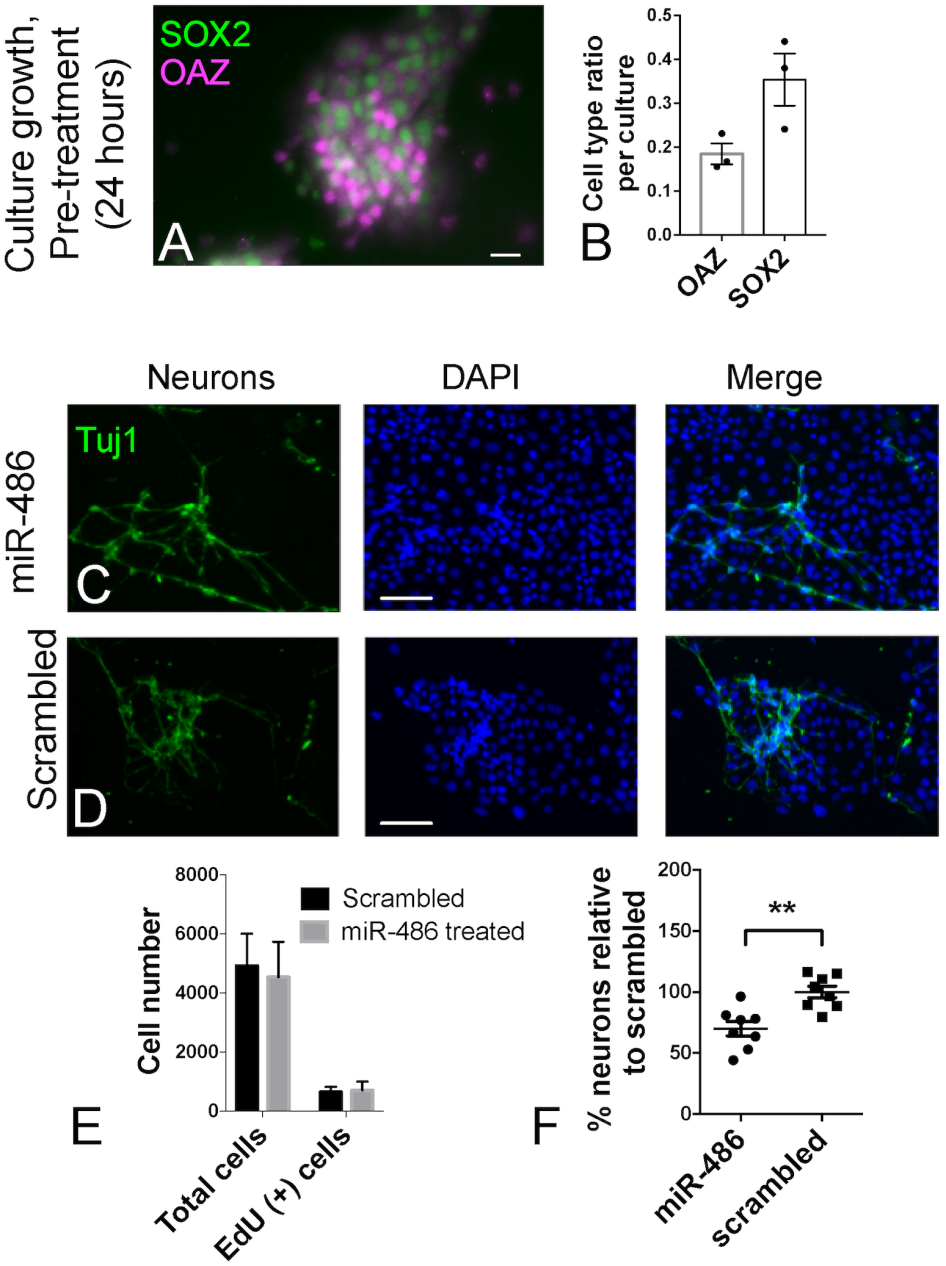
Functional effects of miR-486 in c-Kit-sorted progenitor basal cell culture preparations. (A, B) Basal cells purified from OE of regenerating mice were seeded for short primary culture assay. Characterization of cultures at 24 hours confirms the presence of OE progenitors. Antibody against the neuron-specific transcription factor OAZ (magenta) labels the nuclei of neuron-committed GBCs and nascent or immature neurons; antibody against SOX2 (green) labels nuclei of cells scattered in basal cell islands, consistent with the growth of “upstream” undifferentiated GBCs. Culture composition is quantified in (B), n = 3. (C, D) Cultures were transfected after 24 hours with either miR486 or a scrambled control RNA. 48 hours post transfection, cells were fixed and stained for TUJ1 and DAPI and subsequently quantified. Although there was no significant change in proliferation by EdU incorporation (E), miR-486 treatment reduced the total amount of TUJ1 positive neurons by 30.05% ± 5.88 (p = 0.0014) normalized to scrambled control group (F); n = 8, mean ± S.E.M; student’s t-test. Bar = 20μm in A, 120μm in C and D.

In an effort to identify a function for miR-486 during olfactory neurogenesis, we next performed *in vitro* culture assays. Adult mouse globose basal cells, purified based on c-Kit expression as described above, were placed in short term culture [31]. Immunoselection for the OE basal cell marker was used to minimize seeding cultures with lamina propria contaminants, such as fibroblasts or mesenchymal-like cells. When cultured primarily, late embryonic globose basal cells readily produce neurons over 2–3 days [39]. Here, we used a 3-day culture system seeding adult mouse basal cells harvested from regenerating OE, since miR-486 was identified from adult OE tissue. Cultures were characterized by fixing cells after 24 hours and processing for immunocytochemistry. We used OE cell type-specific markers, as described for staining of tissue sections in Fig 3. As reported previously, adult OE basal cells form adherent undifferentiated-appearing islands when seeded on vitronectin substrate in a neural stem cell medium [31]. We found here that after 24 hours, culture islands contained many SOX2 (+) cells, consistent with expansion of OE basal progenitor cells (Fig 4A and 4B). Probing cultures for expression of the neuron lineage-specific transcription factor OAZ (Fig 4A and 4B), we found that these early basal cell islands contained 18±2% OAZ (+) cells (n = 3 cultures, mean±SEM). The staining pattern identified here confirms that the primary short-term cultures contain OE basal progenitors, and many of the cells express the early olfactory neuronal marker OAZ. Moreover, when analyzed after an additional 48 hours, abundant Tuj1 (+) process-bearing cells are present (Fig 4C). Therefore, the cultures are suitable for analyzing OE neurogenesis in vitro.

Cultures were used to assay for the effect of over-expression of miR-486 (Fig 4C–4F). Comparing cultures transfected with either miR-486 or a scrambled control (n≥3 samples per group), we quantified cell number, neurons, and incorporation of a 1 hour pulse of EdU to assess proliferation. Over-expression was validated by qPCR (Fold Change = ~20,000; p<0.0001). No difference in EdU labeling was identified, comparing scramble or miR-486 treated cultures (Fig 4E). Neurons were readily identifiable based on morphology as cells bearing long neurites extending from a round soma, with expression of neuron-specific β-tubulin, recognized by the Tuj1 antibody. To quantify only convincingly neuronal cells, we did not include Tuj1 (+) cells if neurite-like processes were not evident. Of interest, all cultures produced Tuj1 (+) neurons; however, we repeatedly identified approximately a 30% reduction in the percentage of neurons arising in the miR-486-transfected cultures (p = 0.0014), normalizing to control cultures (Fig 4F). Thus, in this assay, miR-486 over-expression reduces neurogenesis. We interpret these results as consistent with a possible feedback regulatory role for miR-486 on olfactory precursor cell differentiation, since no effect on proliferation was identifiable.

## Discussion

We present here a global miRNA profile focusing on the process of adult olfactory neurogenesis. Using a mouse olfactory lesion-regeneration model, we identified 10 miRNAs enriched in the progenitor population and 28 miRNAs enriched in the non-progenitor cell pool. Furthermore, miR-486, the most highly enriched miRNA in the non-progenitor pool, was chosen for additional study. We found that miR-486 was expressed in OE neurons by histologic analysis, and miR-486 inhibited neuron production in a primary culture over-expression assay. While miR-486 has been reported as highly expressed in blood cells [40, 41], our results on miR-486 expression in neuronal cells are supported by two other reports that found miR-486 expressed in motor neurons [42] and iPSC-generated dopaminergic neurons [41, 43]. Our functional results are in line with a study showing that up-regulation of miR-486 following spinal cord injury promoted neurodegeneration [42].

Regulatory roles for non-coding RNAs, including miRNAs, are an active area of study in many biologic processes. Given the broad importance of miRNAs in post-transcriptional gene regulation during vertebrate neural development [44], it is not surprising that they are involved in regulating adult neurogenesis. Indeed, prior studies have identified miRNA expression in developing OE and found that broadly deleting miRNA production in OE progenitors perturbs OE development [10]. However, the prenatal lethality of that approach precluded further assessment of adult mammalian olfactory tissue maintenance or regeneration. The experiments described here were designed to begin to address whether and how miRNAs may be involved in the maintenance of adult OE. During development, the miRNA-200 family was previously found to be important in OE neuron differentiation [10]. Of interest, key targets known to be regulated by this family include Polycomb complex proteins SUZ12 and BMI1, which have been recently found to be involved in adult OE neurogenic lineages [31]. Given that many acquired human olfactory losses, such as presbyosmia (age-related olfactory decline) or post-viral olfactory disorder (a poorly understood condition that may result in permanent impairment), are associated with histopathologic evidence of neurogenic exhaustion [25, 45], a better understanding of the processes controlling adult OE maintenance is of interest.

MiR-486 was identified by our microarrays as highly enriched in the c-Kit (-) non-progenitor cell fraction. Therefore, we chose this miRNA for further validation studies and confirmed these findings using qPCR and *in situ* hybridization. Using an *in vitro* functional assay, we found that miR-486 over-expression suppressed the neuronal differentiation of basal progenitor cells. Interestingly, our results are in line with a report showing that up-regulation of miR-486 following spinal cord injury promoted neurodegeneration, by suppressing NeuroD6 [42]. In the mouse OE, NeuroD has been found to function in the immediate neuronal precursor stage GBCs, which directly produce new olfactory neurons [46]. As such, it is possible that miR-486 could function analogously in the OE, via interacting with NeuroD. In addition, we have demonstrated that a short-term primary cell culture assay, using purified adult c-Kit (+) basal cells, is useful for further testing of miRNAs of interest. Among the panel of differentially expressed miRNAs identified here is miR-146a. MiR-146a was also reported as down-regulated in olfactory mucosa mesenchymal stromal cells [47]. Understanding interactions between cells of the nasal lamina propria, or mesenchyme, and overlying OE is an area of active investigation, and consideration of potential roles for miR-146a in such interactions is of interest.

Although beyond the scope of the present study, our list of differentially expressed miRNAs identified in regenerating adult OE provides potential therapeutic candidates warranting further investigation. Moreover, in terms of identifying candidates with translational potential, miRNA-mimics or anti-miRs may be attractive for therapeutic use and are currently in clinical trials [48]. In humans, the OE is accessible for the delivery of localized topical therapies; further study of modulatory effects of candidate miRNAs in culture assays and *in vivo* mouse models may hold translational potential.

## Conclusions

We show here that several miRNAs are selectively enriched in progenitor or non-progenitor cell fractions in the regenerating adult mouse OE. We validated miR-486 enrichment in the c-Kit (-) population, and found it to have an inhibitory effect on neuronal production in a culture assay. To our knowledge, this is the first report to specifically address miRNAs implicated in mature mammalian OE maintenance. This work can serve as a starting point for investigating the functional and translational potential of these miRNAs.
